# Supplementary material for: Determining Factors for Pertussis Vaccination Policy: A Study in Five EU Countries
Source: Vaccines (Basel). 2020 Jan 26;8(1):46. doi: 10.3390/vaccines8010046 (PMC7158661; doi:10.3390/vaccines8010046)
Supplement: Supplementary file 1 [file vaccines-08-00046-s001.pdf]

Supplementary Table S1. Pertussis Vaccination Policy in 14 EU Countries since Introduction. [3,4,6,16–50]

Country	Year	Vaccine	Prime Schedule	Booster (Childhood)	Booster (Teenage)
Belgium	1950s	wP	3/4/5/13 month	–	–
	1999	DTaP; wP in FR	–	–	–
	2001	DTaP-IPV	–	–	–
	2003	–	2/3/4/15 month	–	–
	2004	DTaP-IPV-Hib/HB	–	4–6 year DTaP-IPV	–
	2009	–	–	–	14–16 year dTap
Czech	1958	wP	0/6 week/6/18–20 month	3/6 year wP	–
	1991	DTwP	9/13/17 week/18–20 month	5year DTwP	–
	2007	DTaP	9/13/17 week/18–20 month	5–6 year DTaP	–
	2009	–	–	–	10–11 year DTaP-IPV
	2016	DTaP-IPV-Hib/HB	3/5/11–13 month	–	–
Denmark	1961	DTwP	5/6/7/15 month	–	–
	1969	wP	5 wk/9 wk/10 month	–	–
	1997	DTaP-IPV	3/5/12 month	–	–
	2002	DTaP-IPV/Hib	–	–	–
	2003	–	–	5 year dTaP	–
	2004	–	–	5 year dTaP-IPV	–
Finland	1952	DwP	3/4/5 month	–	–
	1957	DTwP	–	3–4/6–7 year DTwP	–
	1977	–	3/4/5/24 month	–	–
	2003	–	–	6 year dtaP	–
	2005	DTaP-IPV-Hib	3/5/12 month	–	–
	2008	–	–	4 year DTaP-IPV	–
	2009	–	–	–	11–13 year dtaP
	2011	–	–	–	14–15 year dtaP
France	1959	wP	3/4/5/18–24 month	–	–
	1966	DTwP-IPV	3/4/5/18–24 month	–	–
	1995	DTwP-IPV-Hib	2/3/4/16–18 month	–	–
	1998	–	–	–	11–13 year DTaP-IPV
	2004	DTaP-IPV-Hib	–	–	–
	2008	DTaP-IPV-Hib (HB)	–	–	11–13year dTaP-IPV
	2013	–	2/4/11 month	6 year DTaP-IPV	–
Germany	1964	wP in GDR	–	–	–
	1969	wP in FWG	–	–	–
	1974	Nil in FWG	–	–	–
	1991	DTwP in all	3/4/5/24 month	–	–
	1995	–	3/4/5/13 month	–	–
	2000	–	2/3/4/11–14 month	–	9–17 year dTaP
	2006	–	–	5–6 year dTaP	
Ireland	1952	DTwP	–	–	–
	1995	DTwP	2/4/6 month	–	–
	2001	DTaP-IPV-Hib	–	–	–
	2008	DTaP-IPV-Hib/HB	–	–	–
	2010	–	–	4–5 year DTaP	–
	2012	–	–	–	12–13 year dTaP
	2016	–	–	–	–
Italy	1961	wP	3/5/11–12 month	–	–
	1995	DTaP	3/5/11 month	–	–
	1999	–	–	5–6 year DTaP	–
	2012	–	–	–	11–18 year dTaP
The Netherlands	1957	DTwP	3/4/5 month	–	–
	1962	DTwP-IPV	3/4/5/11 month	–	–
	1993	DTwP-IPV, Hib	–	–	–
	1999	–	2/3/4/11 month	–	–

	2002	–	–	4 year aP	–
	2003	DTwP-IPV-Hib	–	–	–
	2005	DTaP-IPV-Hib	2/3/4/11 month	–	–
	2006	–	–	4 year DTaP-IPV	–
	2008	–	–	–	–
	2010	–	–	–	–
	2011	DTaP-IPV-Hib/HB	–	–	–
	2017	–	–	4 year dTaP	–
	1952	DTwP	3/4/5/15–18 month	–	–
	1984	–	3/5/10 month	–	–
Norway	1998	DTaP-IPV/Hib	–	–	–
	2006	–	3/5/12 month	7 year DTaP-IPV	–
	2012	–	–	–	15 year DTaP-IPV
	1960	DTwP	2/3–4/5/16–18 month	–	–
Poland	2004	–	–	6 year DTaP	–
	2016	–	–	–	14 year dTaP
	1965	wP	–	–	–
	1967	–	–	–	–
	1975	–	3/5/7 month	–	–
	1995	DTwP	2–3/4–5/6–7/18 month	–	–
Spain	2000	–	2/4/6/18 month	4–6 year DTaP	–
	2005	DTaP	–	–	–
	2012	–	–	4–6 year DTaP/dTaP	11–14 year dTaP
	2014	–	–	–	11–12 year dTaP
	2015	–	–	6 year DTaP/dTaP	–
	2016	DTaP-IPV-Hib/HB	2/4/12 month	6 year dTaP-IPV	–
	1953	DTwP	3/5/12 month	–	–
	1979	Nil		–	–
	1996	DTaP	3/5/12 month	–	–
Sweden	1998	DTaP-IPV/Hib	3/5/12 month	–	–
	2005	–	–	–	10 year DTaP till 2011
	2007	–	–	5–6 year DTaP-IPV	–
	2016	–	–	–	14–16 year dTap
	1957	DTwP	–	–	–
	1968	–	3/5/11 month	–	–
	1990	–	2/3/4 month	–	–
UK	2000	(DTaP3-Hib)	–	–	–
	2001	(DTaP3-Hib)	–	3 year 4 month DTaP-IPV	–
	2004	DTaP-IPV-Hib	2/3/4 month	–	–
	2017	DTaP-IPV-Hib/HB	–	–	14 year dT-IPV

Supplementary File S2. Semi-Structured Interview Topic Guide

First of all, thank you for taking the time to meet with me today. My name is ____________; I am conducting interviews with key informants for the project titled, "Pertussis vaccination in Europe: Determining factors of vaccine type and schedule for childhood immunisation programme and recommendation for pregnant women."

In this interview, I am going to ask you some questions about pertussis immunisation in your country. The interview should take about 30 min. I want to make sure I have captured your comments accurately; therefore, I will be recording this interview and use the tape to produce a transcript. This transcript will be sent back to you to check and sign as an endorsement of accuracy. When I receive the endorsed transcript from you, I will delete the audio-file, is that ok?

(1). What is the type of pertussis vaccine being used in the childhood immunisation programme in (country) at the moment? Has this always been used in (country)? If not, what was the previous vaccine used? (Whole cell/acellular monocomponent/acellular 2 or 3 or 5 antigens).
(2). What could be the reasons of using (or changing to) this type of vaccine in (country)? What do you think are the advantages and disadvantages of this vaccine?
(3). What is the current schedule of pertussis childhood immunisation? Is it mandatory or recommended?

(4). How does it differ from the previous immunisation scheme? Can you think of the reasons for such change?
(5). In your recollection of the pertussis vaccination policy, can you think of any other changes that have taken place, either in its content or delivery?
(6). Why do you think it was important for (country) to implement this change?
(7). What is the coverage of the current programme? Is the national immunization programme well-received?
(8). Currently, is there any recommendation for parents regarding pertussis vaccination? If so, what is it? (cocooning/vaccination in pregnancy).
(9). Why do you think it is important for (country) to recommend (cocooning/vaccination in pregnancy) instead of (vaccination in pregnancy/cocooning)?
(10). Do you agree with this recommendation? Would you have followed the recommendation if you were expectant parents or grandparents? Why?
(11). Do you know what is being debated in (country) at the moment? Is there any policy or recommendation planned for the future?
(12). Is there anything you would like to add, anything you think that is important but I haven't asked you?

Supplementary File S3. Coding Guideline

1. Read the transcript quickly to get an impression of the whole picture of the discussion.
2. Read the codebook carefully to familiarise yourself with the categories and codes.
3. Start coding:
    a. Read the whole chunk between/and grasp the central idea or main argument.
    b. See if it answers our question: "What factors determine vaccine policy?" and if so, which reasons (codes from Cats. A, B and C) apply.
    c. See if it describes a policy or strategy (Cat. D) a phenomenon (Cat. E), or if it criticises the evidence aspect (Cat. F).
    d. The primary code (1st code you put down) should be as close to the central idea or main argument as possible; it should be a code that best summarise the whole chunk so think parsimoniously.
    e. You can put down more than one code to each chunk; the other codes (2nd, 3rd…etc.) should aim to tag any concepts that are relevant and to be thorough.
    f. If you think no code is suitable and you want to create a code, you can add new codes. Here are two example:

> /I am not sure what I am talking about, let's say, as the policy maker of country U, I am worried about terrorist attack via pertussis as bioweapon and unlike GPs, I don't care that much about safety profile, I think mandatory vaccine is the way to go./

Primary code: "different priority of stakeholders".
Other codes: "mandatory vaccine", "concern of national security".

> /I believe teenagers should be given a voice too as they have no participation in the vaccination policy process but I would say, teenagers nowadays are not like before, look at Greta Thunberg, shouldn't we consider their choice too? /

Primary code: "teenagers' perspective".
Other codes: "teenagers nowadays deemed more knowledgeable".

You can just add new codes as below and we will have a meeting afterwards to talk about new codes:

X1 concern of national security
X2 teenagers' perspective
X3 teenagers nowadays deemed more knowledgeable

A Scientific/Technical Reasons:

1. AEFI type & severity/safety profile
2. AEFI frequency
3. efficacy (textbook effect)
4. effectiveness (real world effect)
5. prevent disease (symptoms)
6. prevent infection (state of carriage, contamination, with or without symptoms)
7. prevent transmission (spread from infected to uninfected)
8. adult reservoir/circulation in population
9. duration of protection
10. level of protection
11. secondary vaccine failure i.e., waning immunity
12. cases (infants, children)
13. cases (adults)
14. infant death
15. non-fatal consequence (sequelae, hospitalisation)
16. technical process (production of antigens, detoxification)
17. immunity response (Th1, Th2, Th17)
18. immunity blunting

B Logistical Reasons:

19. marketing authorisation/SPC/labelled indication
20. driven by consumption/demand
21. driven by production/supply
22. infrastructure breakdown
23. economic difficulties/price & cost
24. cost-effectiveness
25. tender process
26. availability of combined vaccine
27. lack of mono-valent vaccine

C Sociological Reasons:

28. public acceptability towards vaccine & policy
29. social sensitivity towards AEFI
30. attitude & behaviour of HCP
31. attitude & behaviour of pregnant women/parents
32. historical event/vaccine scares
33. media coverage & social media
34. public emotion
35. low awareness of pertussis/not perceived as dangerous
36. religious grounds
37. interest (econ, political, fame) of specific groups
38. constitutional/bioethical consideration
39. trust in health care professionals
40. trust in government/state public health agency
41. sentiment/grudge due to AEFI being dismissed
42. incentive of protecting mother
43. incentive of protecting baby
44. incentive of herd immunity/solidarity
45. vaccine advocates (convinced, accept, mobilised)
46. vaccine critics (radical, refusal, antivax)
47. vaccine critics (reformist, partial aceptance)

48. vaccine hesitants (doubtful, undecided, delayed acceptance)

D Strategies, Policy & Decision Process:

49. preferential recommendation
50. mandatory vaccination
51. voluntary/optional/a la carte vaccination
52. philosophical exemption of mandated vaccine
53. emergency/outbreak measure
54. financed by self
55. financed by state/reimbursement
56. securing timely vaccination
57. booster (children, adolescent, cocooning strategy)
58. ridiculing/stigmatisation of antivax
59. consulting experts/advising committee
60. consulting citizen
61. referencing neighbour countries/intl. agencies

E Phenomena:

62. trade-off (individual) = safety vs. efficacy
63. trade-off (population) = coverage vs. protection
64. different priority of stakeholders
65. (medics, parents, epi, Ministry of Health, Ministry of Education, public)
66. consensus among stakeholders
67. choice of public vs. private health care
68. social structural change (grandparents contact, sibling contact)
69. social differentiation in vaccine hesitancy (more in middle-class, higher edu, urban)
70. discordant vaccine attitude in family
71. info spread on Facebook emphasise on extremes
72. concern of pockets of severely under-vaccinated
73. concern of polarising attitude

F Quality of evidnce:

74. inappropriate comparison of aP & wP
75. change in surveillance method
76. change in diagnostic method
77. little attention & output in social research
78. med & vac experts may not have social science expertise

X. New codes: (to be added by coder)
